# Nano polarimetry: enhanced AFM-NSOM triple-mode polarimeter tip

**DOI:** 10.1038/s41598-020-72483-9

**Published:** 2020-10-01

**Authors:** Matityahu Karelits, Zeev Zalevsky, Avi Karsenty

**Affiliations:** 1 Department of Applied Physics/Electro-Optics Engineering, Advanced Laboratory of Electro-Optics (ALEO), Jerusalem College of Technology (JCT-Lev Academic Center), 9116001 Jerusalem, Israel; 2 Bar-Ilan University, Faculty of Engineering, 5290002 Ramat Gan, Israel; 3 Nanotechnology Center, Bar-Ilan University, 5290002 Ramat Gan, Israel; 4 Nanotechnology Educational and Research Center, Jerusalem College of Technology, 9116001 Jerusalem, Israel

**Keywords:** Silicon photonics, Atomic force microscopy, Nanoscale devices, Nanosensors

## Abstract

A novel application of a combined and enhanced NSOM-AFM tip-photodetector system resulted in a nanoscale Polarimeter, generated by four different holes, each sharing a different shape, and enabling that the four photonic readouts forming the tip will be the four Stokes coefficients, this in order to place the polarization state in the Poincare sphere. The new system has been built on standard Atomic Force Microscope (AFM) cantilever, in order to serve as a triple-mode scanning system, sharing complementary scanning topography, optical data analysis and polarization states. This new device, which has been designed and simulated using Comsol Multi-Physics software package, consists in a Platinum-Silicon drilled conical photodetector, sharing subwavelength apertures, and has been processed using advanced nanotechnology tools on a commercial silicon cantilever. After a comparison study of drilled versus filled tips advantages, and of several optics phenomena such as interferences, the article presents the added value of multiple-apertures scanning tip for nano-polarimetry.

## Introduction

### Surface scanning background and needs

With the progress in nanotechnology and materials analysis, the investigation in surface scanning domain becomes more complex and challenging. If in the past, one scanning approach was good enough to evaluate the behavior of particles on a sample’s surface, today it appears that combining several techniques became a “must” and not anymore a “nice-to-have” useful tool. Across the last decades, the core methods were developed separately: The Atomic Force Microscopy (AFM) as the core method of Scanning Probe Microscopy (SPM) for nanoscale characterization of surface topography and morphology, and the Near Field Scanning Optical Microscopy (NSOM), as a sub-diffractive optical characterization method. One can intuitively understand that these two core methods can serve as complementary analyses in a case study of characterizing fluorescence or electroluminescence of nanoscale structures. Since it was discovered few decades ago^1^. NSOM remains one of the optics most challenging domains. These complementary methods are widely used for nanoscale study and characterization of new nanomaterial components^2^, biological objects^3^, plasmonics probing^4^, differential NSOM^5^, and more. They have usually used separately^6^ or additionally^7^. If the market is large, still there are less than twenty main worldwide key players/manufacturers in this Scanning-Probing market^8^, where some of them are Key Players in AFM^9^ and NSOM^10^ separated technologies. Since there is a constant growing need to conduct advanced research on biological and non-biological material, the need for smart innovative solutions conducted manufacturers to seek for new kind of equipment, integrating leading technological features and well equipped microscopes. In our article we present the added values of a combined device enabling a triple-mode usage (topography, optical, and polarization) of both AFM and NSOM measurements, when collecting the reflected light, directly from the scanned surface while having a more efficient light collection process. Such an analysis may serve as a good forecast of expected behavior and accuracy in measurements.

### AFM-NSOM dual-mode concept

**Figure 1:**
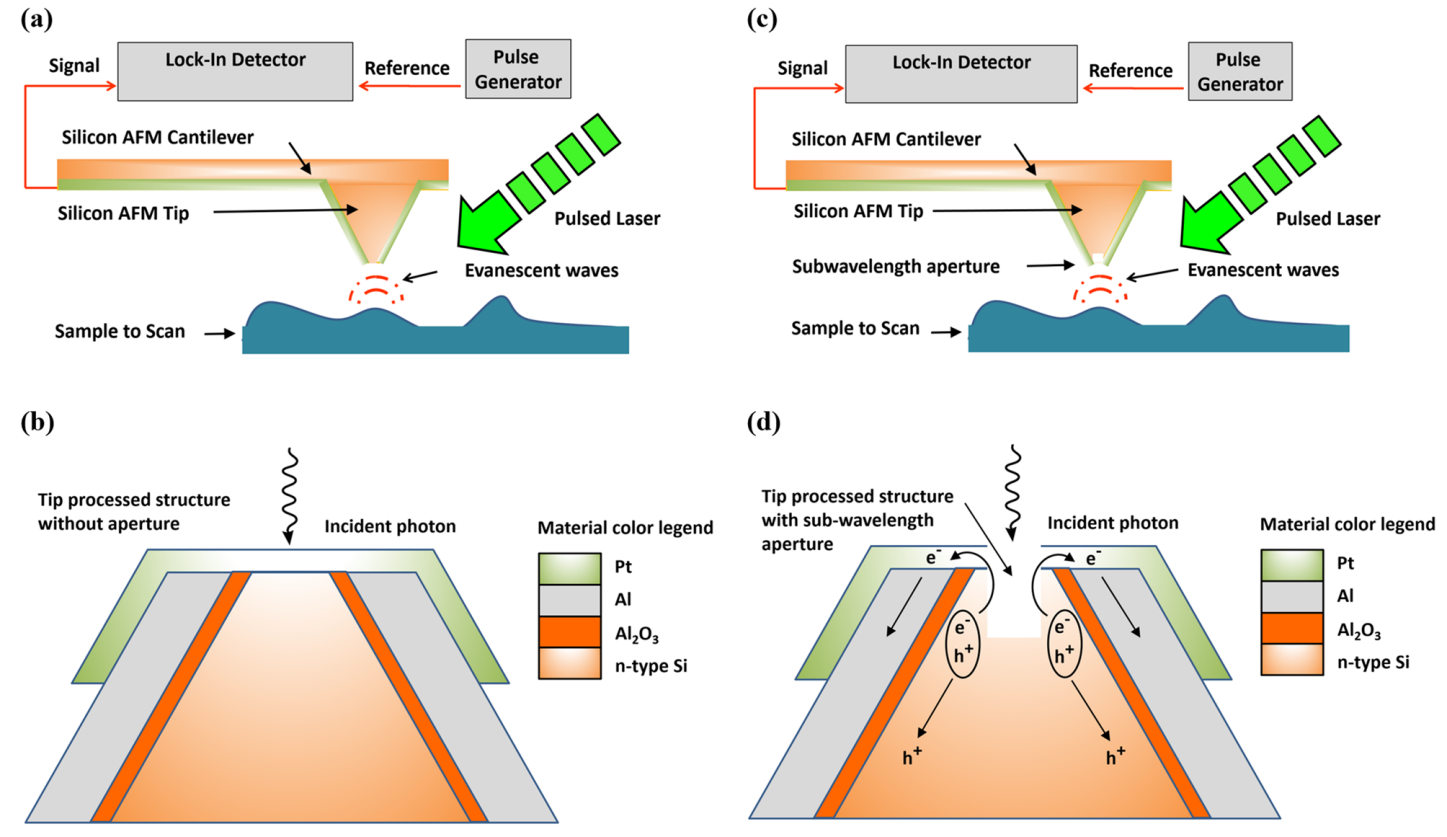
Schematic representation of the AFM-NSOM dual-mode proposed system. (**a**) Concept of the AFM-NSOM coupled device without aperture. (**b**) Concept of the processed tip structure and photo-current generation without aperture. (**c**) Concept of the AFM-NSOM coupled device with sub-wavelength aperture. (**d**) Concept of the processed tip structure and photo-current generation with sub-wavelength aperture.

Before dealing later on with the proposed triple-mode Nano-Polarimeter (AFM, NSOM, and Stokes), combining in one device the capability to collect multiple data in parallel: (1) mechanical topography (AFM), (2) electro-optical info (NSOM), and (3) polarization states (i.e. four apertures), we may progress step-by-step, and first present the combined approach of AFM and NSOM, which have progressed a lot in parallel since the last decades. As a stand-alone analysis, each one of these well-known methods presents solid data analysis advantages. The design and the simulation of new tips towards fabrication^11^, as well as the manufacturing process itself of new tips using new processed methods^12,13^, has largely progressed. With time, the idea of combining several methods was developed and studied, such as the organic light-emitting devices (OLEDs) with micro-machined silicon cantilevers^14^. In our specific case, the idea of combining AFM and NSOM measurement capabilities, into one dual-mode based on silicon tip, is quite unique and presents several advantages and features: dual-mode in one tip, low-cost starting material (standard commercial AFM tip), easy six-steps-based process, energetic efficiency combined with multi-functionality, accurate and direct light acquisition from the sample surface itself, and of course a good SNR (Signal to Noise Ratio). The concept of the improved system is presented in Fig. 1 with two separated configurations: Filled tip-photodetector (Fig. 1a,b), and drilled-tip-photodetector (Fig. 1c,d). In the second configuration, the truncated photo-detector is placed instead the regular silicon tip, at the end of the cantilever (Fig. 1c).

### Filled vs. drilled tip-photodetector

As presented in previous publication^15^, there is a clear advantage in drilled photodetector, since it enables a direct reading from the surface reflection. In this approach, the first advantage is the light collection process, which will be directly performed from the surface of the sample. Some lock-in amplifier can be added to detect the small current signals (in the pA range). As mentioned above, the main advantages are related to the capability of our proposed NSOM-AFM dual mode system to enable both multi-functionality in one device, and also to have increased energetic collection efficiency (due to the fact that reflected light is directly converted to intensity readout at the tip and does not have to be coupled and guided through the collection fiber as done in the case of conventional NSOM system).

## Simulation method and numerical results

### Finite elements method and sensor structure

**Figure 2:**
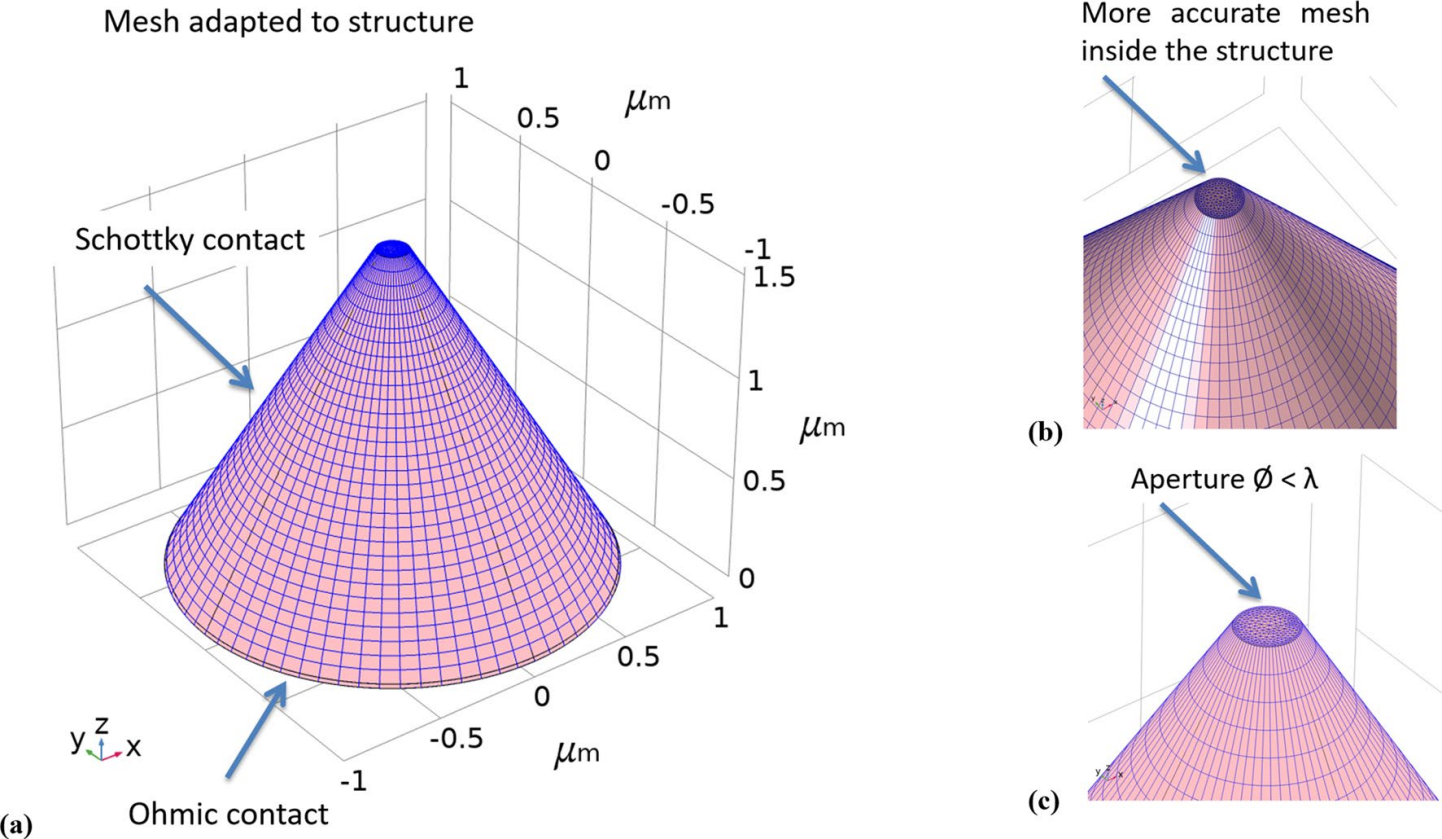
Comsol structure and mesh simulation results of the tip-photodetector. Its main parameters are: Height of 1.6 µm, top diameter of 150 nm, and bottom radius less than 1 µm: (**a**) simulated regular mesh used for external contacts. (**b**) Simulated accurate mesh used for upper inside aperture and zoom-in of the aperture, sharing a diameter of 150 nm only less than the visible wavelength, and of the internal drilled cylinder. (**c**) Zoom-out.

**Figure 3:**
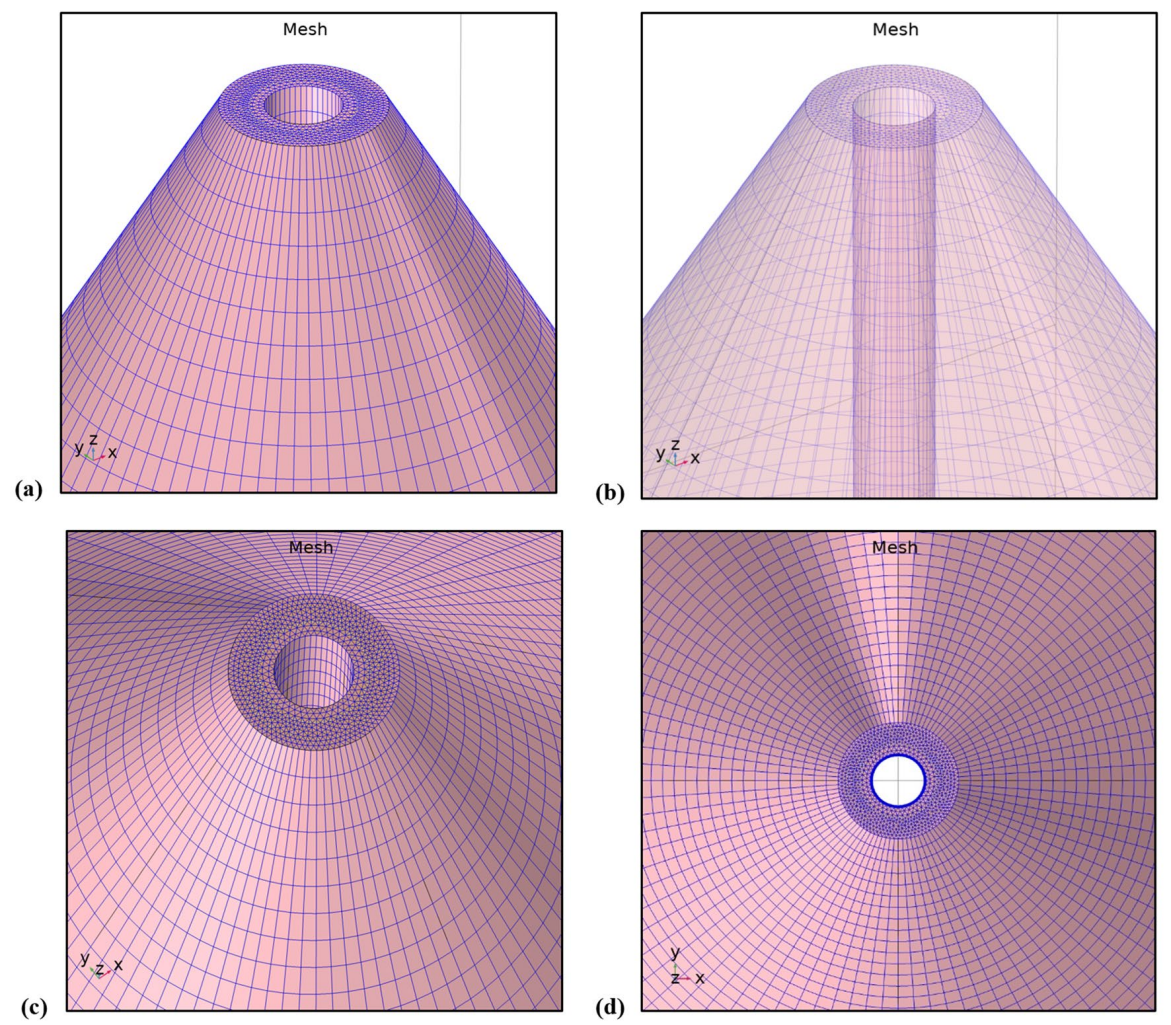
Comsol electrical simulation results of the tip drilled photo-detector at different views. (**a**) Side opaque view. (**b**) Side transparent view. (**c**) Zoom-in of the top mesh. (**d**) Top view.

In order to perform a complete and accurate numerical study of the proposed device, the platform of Comsol Multi-Physics software package^16^ was used. This platform’s approach is based on the Finite Elements Method (FEM)^17,18^. The combined and enhanced NSOM-AFM tip-photodetector system, which we propose, consists of a silicon Schottky photo diode sharing a sub-wavelength aperture at the top of the tip^19^. In such a way, a nanoscale electro-optical sensor, placed on a commercial AFM cantilever’s tip, would enable (1) the collection of the topography (AFM), (2) the collection of the optical data (NSOM), and later on the collection of the four Stokes Criteria (Polarization) when drilling four apertures at its top, creating a triple-mode measurement system, for nano-polarimetry. In the past, electrical and optical responses, were studied and optimized while scanning a laser beam, and high resolution of order of the detector’s aperture was obtained^19^. Since this device structure is quite complex and challenging, in particular in the nanoscale range, the tip-photodetector was first simulated separately, and then combined to simulated standard silicon-based cantilever. As shown below in Figs. 2 and 3, the nano-polarimeter shares a 3D structure of a truncated conical-shaped photodetector device. With a height of 1.6 μm, a top radius of 75 nm (at this stage), and a bottom radius determined by the silicon plane cutting slope, equal to 57° which gives 1 μm for the corresponding height, the device presents several challenges. One of them is the mesh complexity, and therefore the corresponding simulation run time (the more complex the web of elements, the longer the run time). When compared to the commercial tip, sharing a height of 16 μm, the height considered here for simulation was in the range of 1/10 of the real AFM silicon tip’s height.

### Set-up and parameters: concerns and considerations

**Figure 4:**
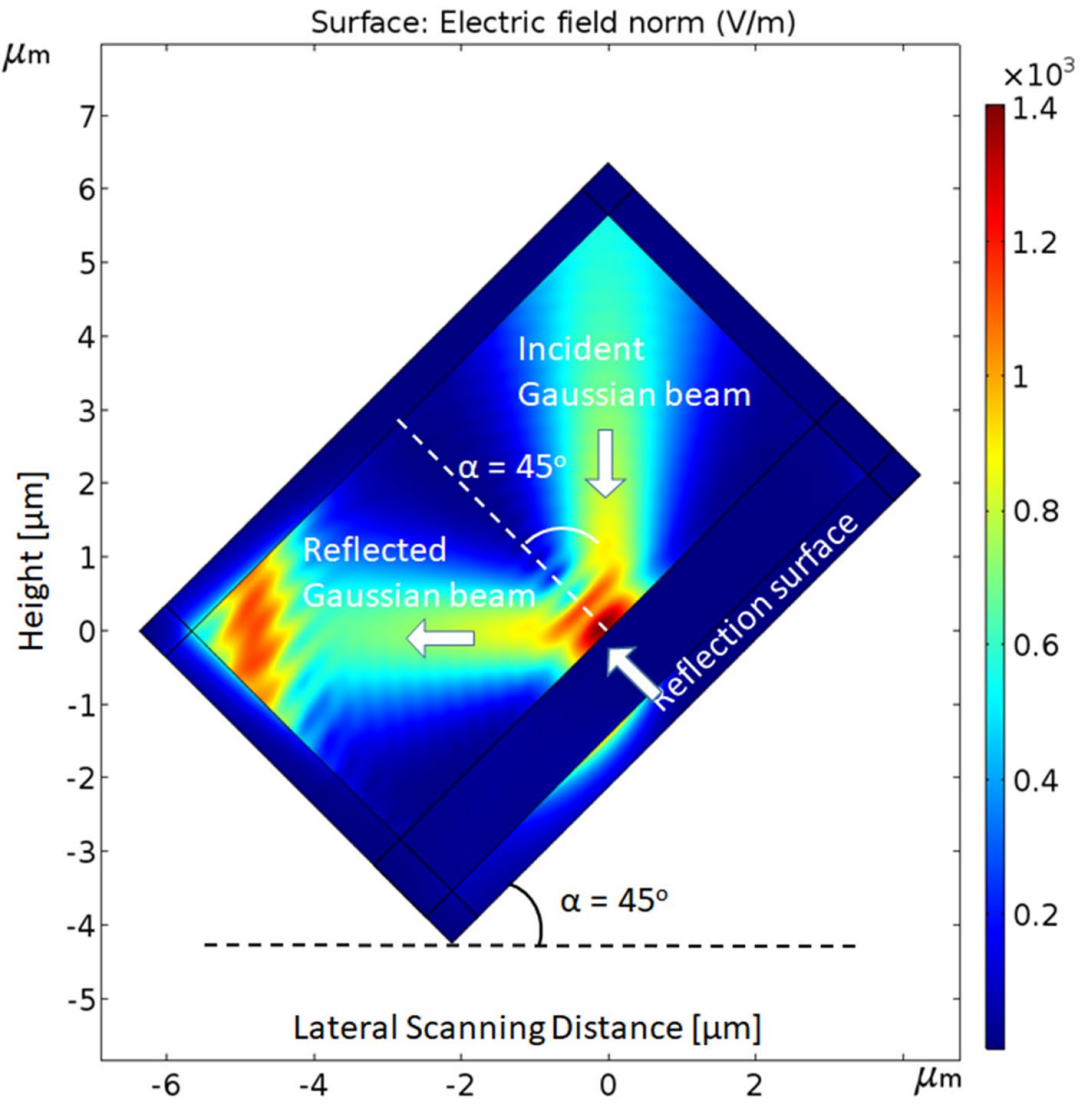
Comsol simulation of the incident and reflected illumination beams interacting with the scanned surface.

The initial set-up for a representative simulation of the reflected light from a surface is presented in Fig. 4. At this stage of the preliminary simulations, no photodetector is added, and the light is directly reflected and captured from the surface. Comsol Multi-Physics Software Package enables to create the simulation inside a virtual box, which serves as the medium of the measurements. For convenience and much more rapid simulations, the box itself was placed in different angles instead of the beam. Of course, there is no limitation to change the angle of the beam on a horizontal surface.

Stepping a leap ahead, the aim of the next following studies was to establish a forecast of the optimized parameters to be used during the scanning of a surface when an incident illumination is reflected with a specific angle, and captured by a photodetector. The difference in the measured photocurrents *ΔI* is presented as a function of several parameters, such as:

- α—the illumination incidence and reflection angle,
- *a*—the height of the photodetector and the scanned surface (contact or proximity mode),
- λ—the wavelength of the illumination,
- *ϕ*—The work function of the photodetector external layer, and more.

**Figure 5:**
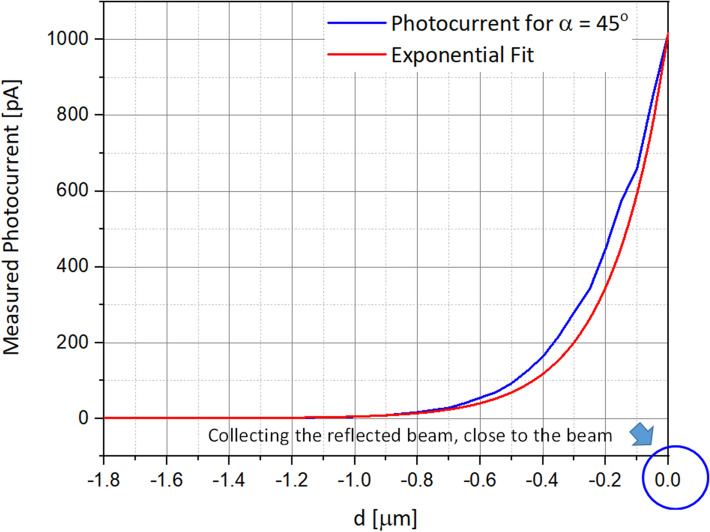
Graph of the measured photocurrent as a function of the scanning distance *d*. As expected, the maximum value is obtained at x = 0, center of the incident illumination.

The chosen parameters are summarized in Table 1 below. In Fig. 5, the representative simulation of the reflected light, directly captured from the surface in the photodetector, is presenting the measured photocurrent for an angle of 45°.

**Table 1:** Parameters used in the simulations.

Parameter	Definition	Value
**Environment set-up parameters**		
*λ*	Wavelength of the illumination	550 nm
*E*	Electric field	× 10^3^ V/m
*φ*	Work function	Al
d	Position of the photodetector relatively to the center	− 2 to 0 µm (symmetry)
a	Distance between the surface and the photodetector	5, 10, 20, 50 nm
α	Incident and reflected angles of the illumination	45°, 60°, 75°
V	Bias negative voltage	− 0.5 V
**Photodetector structure parameters**		
h	Height of the conical photodetector	1.6 μm
R_cylinder_	Radius of the cylinder	0, 15, 30, 45, 60, 75 nm
R_top_	Top radius of the conical photodetector	75 nm
R_bottom_	Bottom radius of the conical photodetector	1 μm
θ	Silicon plan angle	57°

### Photo-current as a function of the illumination angle

**Figure 6:**
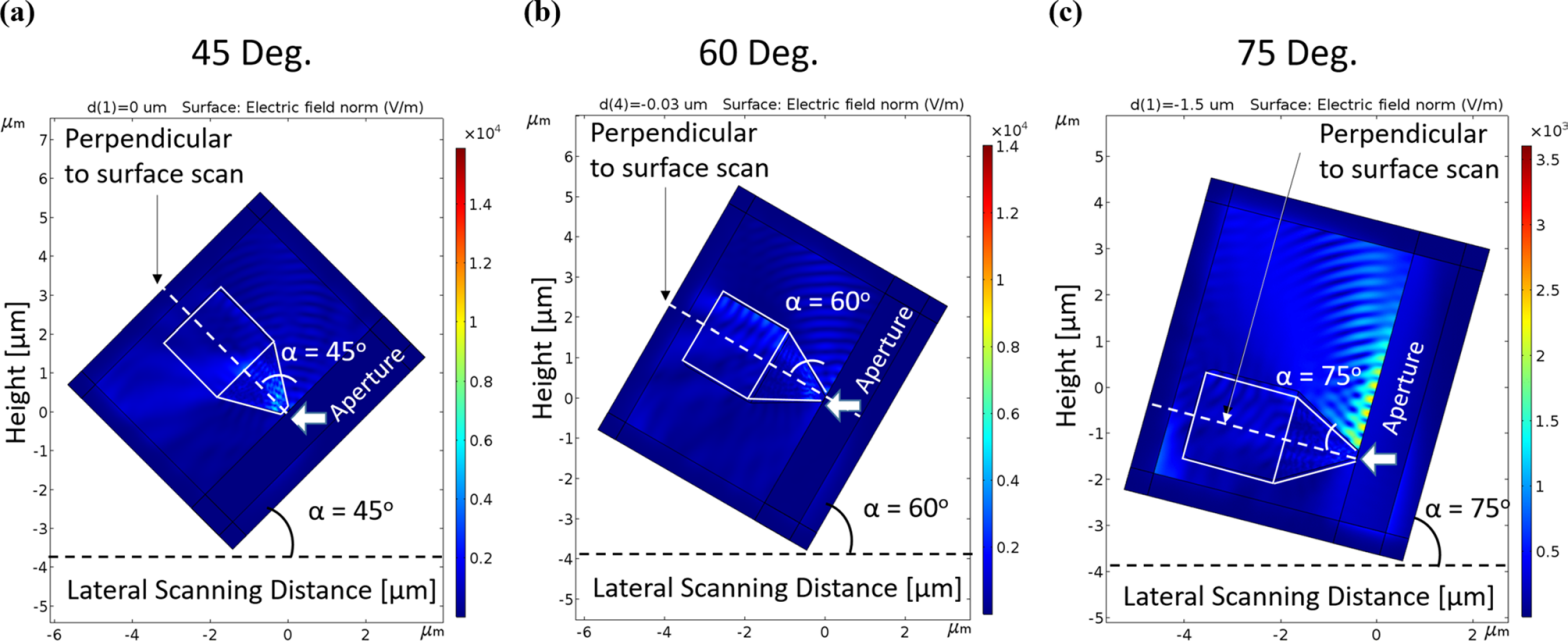
Series of scanning simulations are presenting, when the illumination incident beam angle is varying by steps of 15°, respectively. (**a**) 45°. (**b**) 60°. (**c**) 75°. Again, for simulation purpose only, it was much easier to change the angle of the medium box, instead of the incident beam.

**Figure 7:**
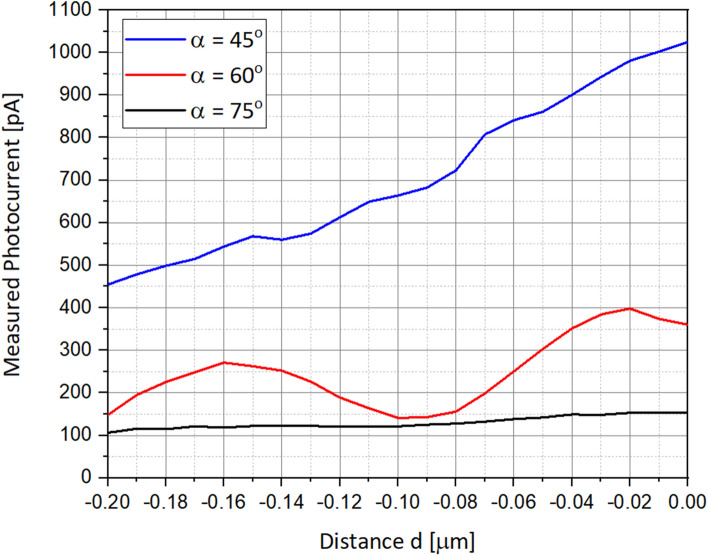
Simulated photocurrents as a function of α, the illumination incident beam angle.

As presented in the two following figures, the next step was to check the influence of the angle of the illumination beam and to identify the optimized one. Figure 6 presents the different angle positions when Fig. 7 presents the corresponding photocurrents.

### Measured photocurrent as a function of the distance from the surface

**Figure 8:**
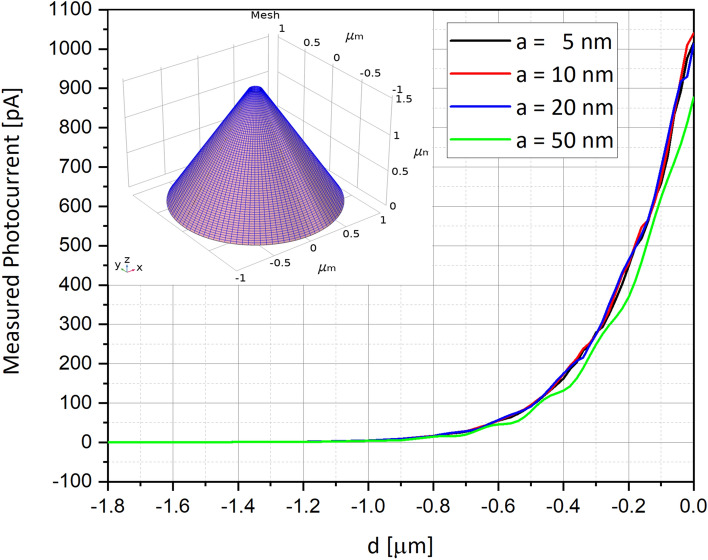
Measured photocurrents for several distances a between the aperture of the photodetector and the scanned surface for filled device. In this case, a filled device was simulated, and the curves are continuous, with small steps of constructive and destructive inferences, mainly in the curve of a = 50 nm. Illumination incident beam angle α is 45°.

**Figure 9:**
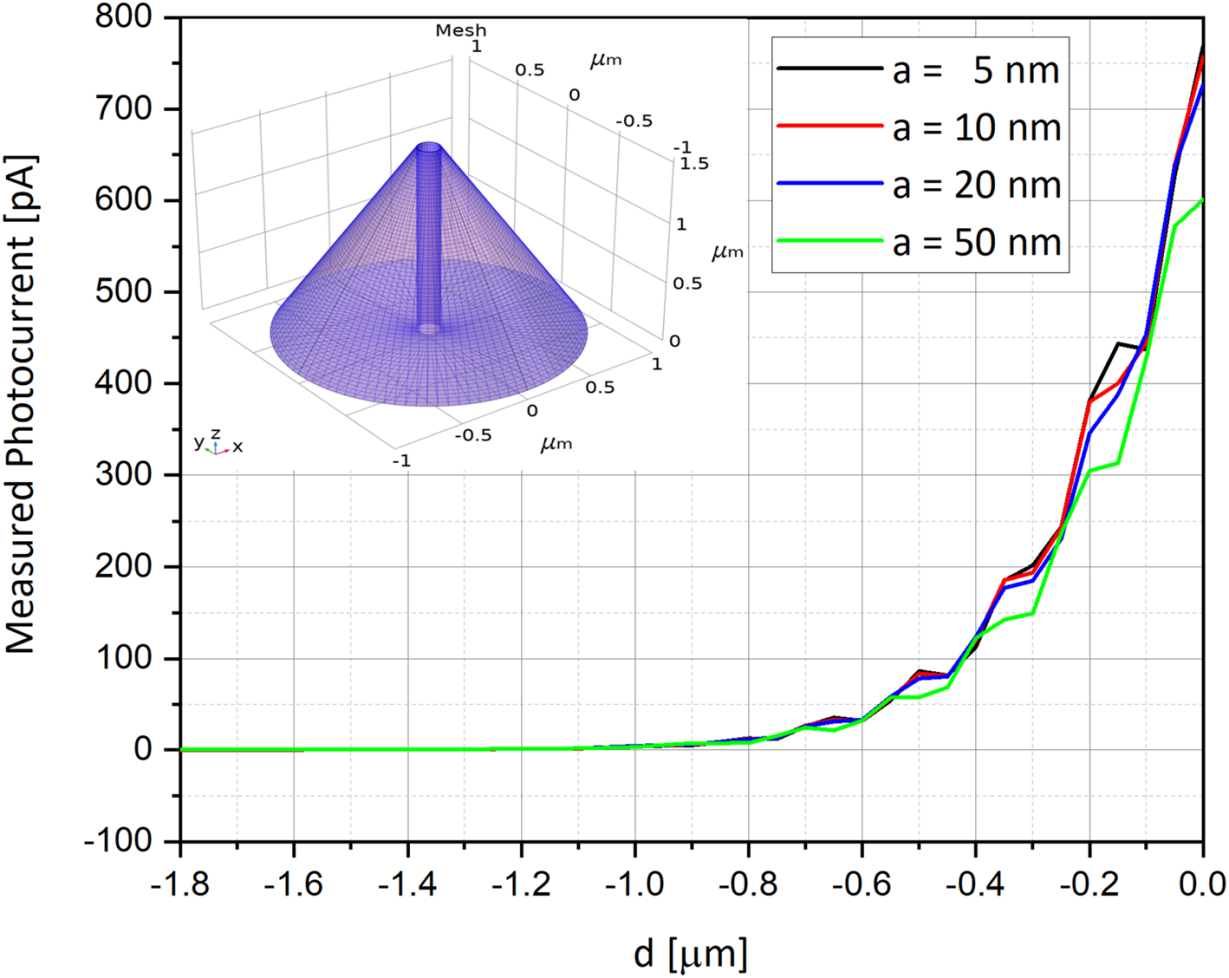
Measured photocurrents for several distances a between the aperture of the photodetector and the scanned surface for drilled device. In this case, a drilled device was simulated, and the curves are continuous, with accentuated steps of constructive and destructive inferences in all the curves, i.e. for all the distances. Illumination incident beam angle α is 45°. Chosen representative radius of the drilled aperture is 35 nm.

**Figure 10:**
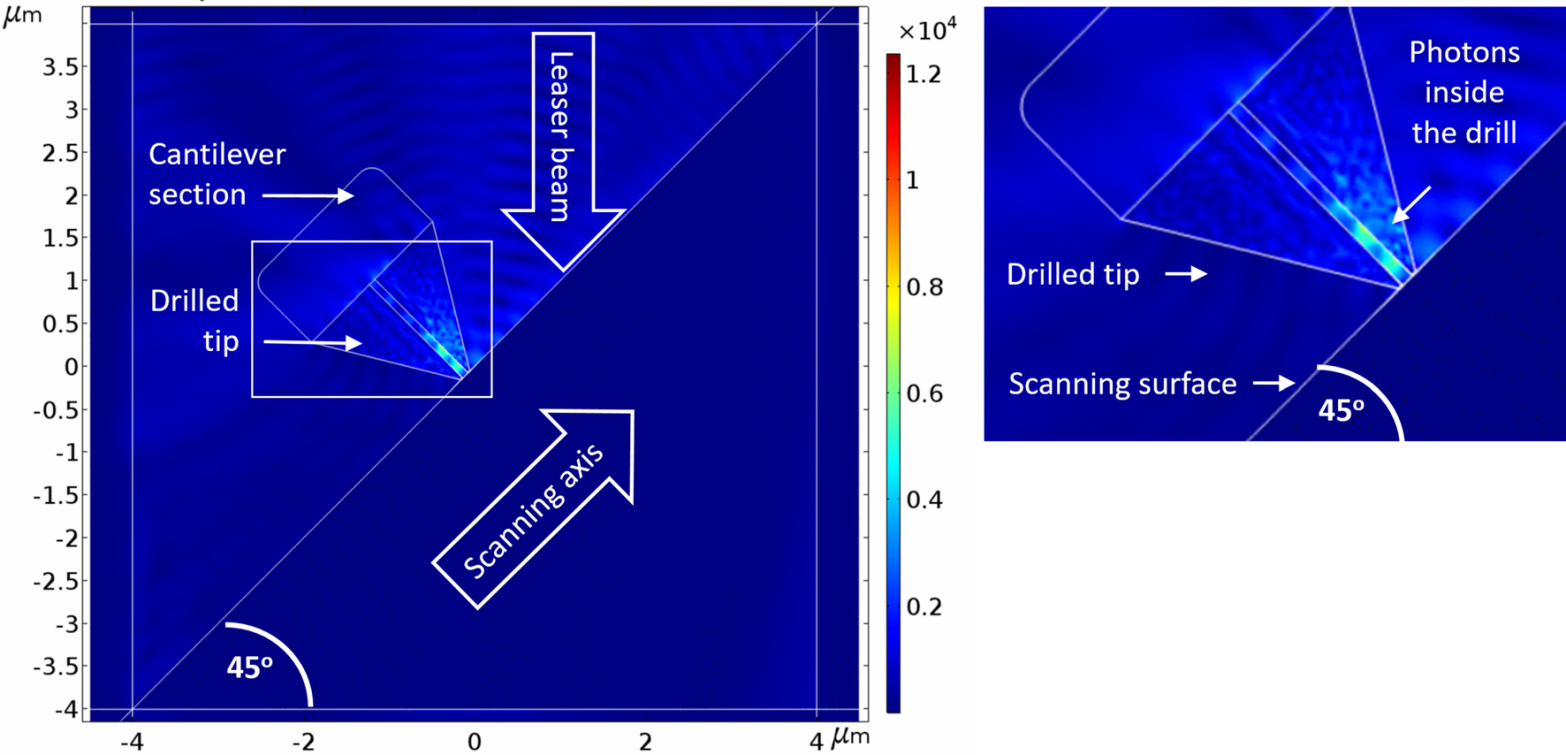
Drilled photodetector scanning the surface in proximity mode. The insert on the right side presents a zoom-out of the photons inside the drilled part of the photodetector. The illumination incident beam angle α is 45°.

Since the new NSOM-AFM photo-detection will be used to scan the surface of materials, it was important to evaluate the optimized distance *a* of the tip from the surface. Series of simulations were performed when varying the distance from 5 nm (almost contact mode) till 50 nm (proximity mode). Two devices were tried and compared, in order to assure the dual-mode existence of AFM and of NSOM capabilities: First, the surface scanning was performed using a filled tip-photodetector (Fig. 8)—e.g. no open cylinder inside the tip as shown in Fig. 1a,b—as a regular AFM tip. In other words, in this case, the check was of the answer of a regular Schottky diode. Then the NSOM mode was checked (Fig. 9), when this time the photodetector, sharing the same dimensions of previous used one, was drilled, as presented in Fig. 1c,d. It was clearly observed that if for regular filled device, the curves are continuous, for the truncated one, the curves present discrete steps. Such steps in current can definitely be interpreted by the amounts of absorbed photocurrent inside the cylinder of the truncated device, but this time with constructive and destructive interferences, as well observed in Fig. 10. Finally, it is important to clarify that for convenience purpose of user friendly simulations, some assumptions were taken in consideration: If the photodetector structures were built in 3D, as shown in the top-left inserts of Figs. 8 and 9, the electro-optical simulations themselves were performed in 2D, as shown in the curves of Figs. 8 and 9.

### Measured photocurrent as a function of the aperture diameter

**Figure 11:**
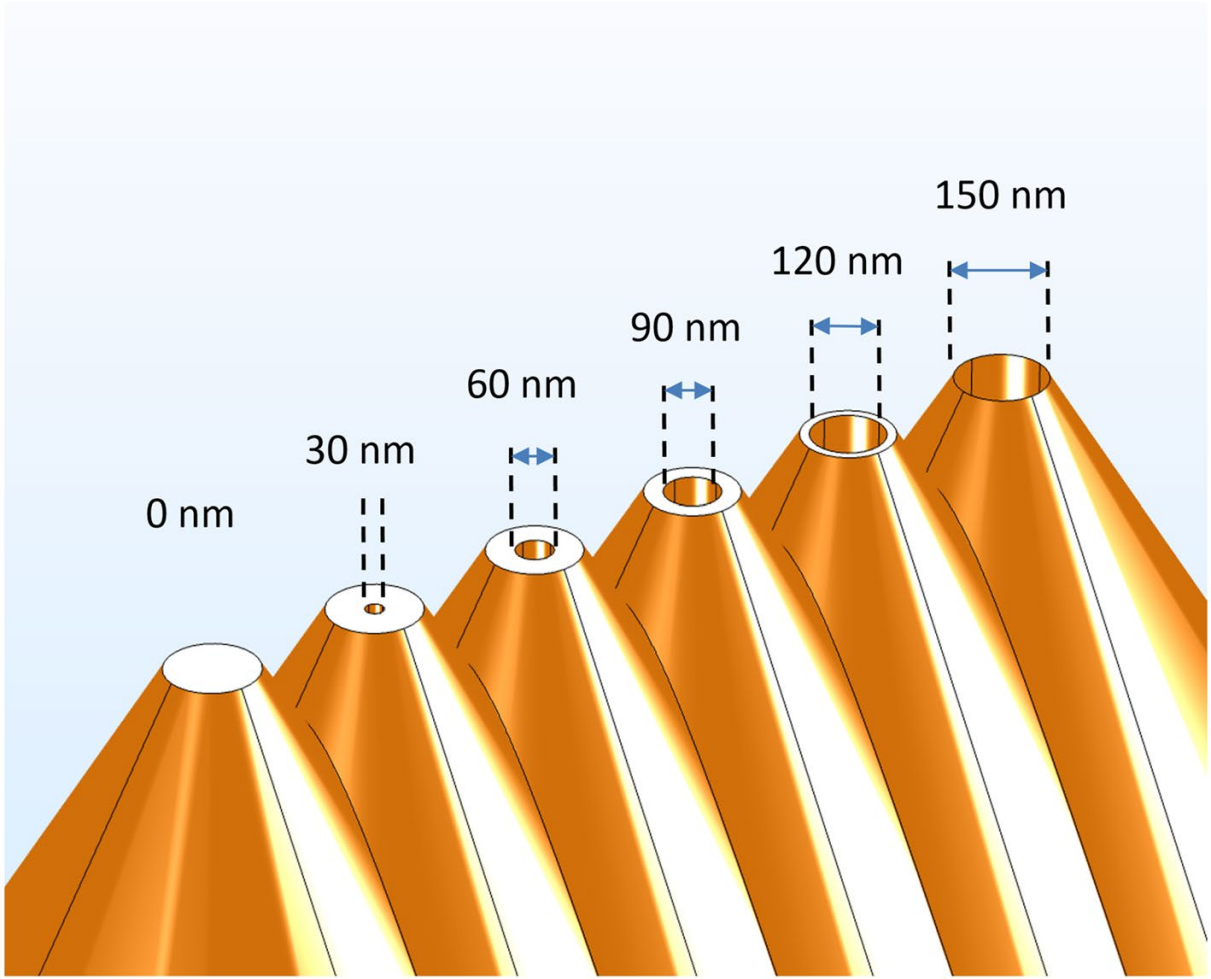
Schematics of series of drilled scanning photodetectors sharing increasing aperture diameters, varying from 0 to 150 nm, by steps of 30 nm.

**Figure 12:**
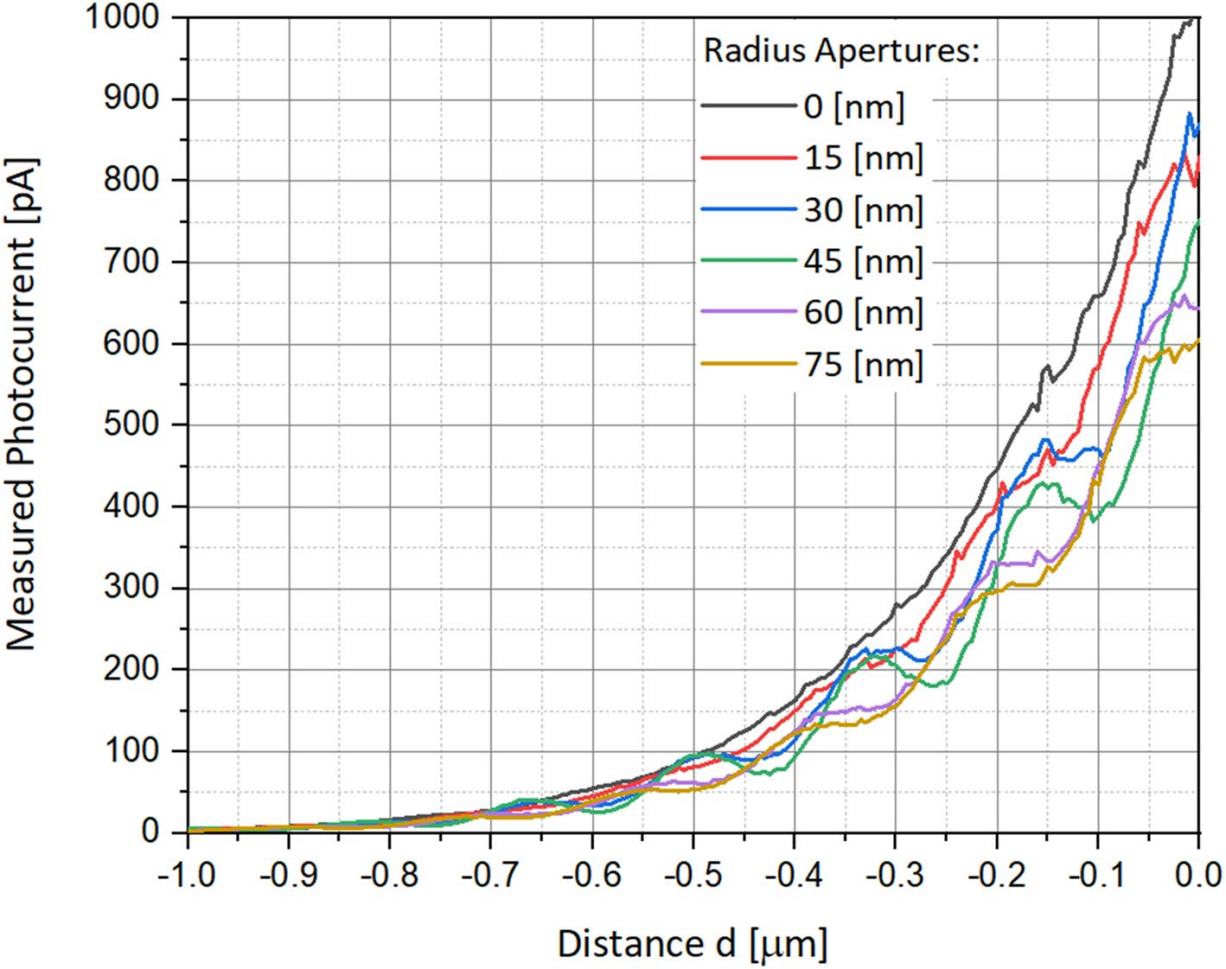
Measured photocurrent as a function of the distance *d*, and for several aperture radii varying between 0 and 75 nm. The distance $a$ is 5 nm, and the angle $\alpha$ is 45°. All other parameters are presented in Table 1.

**Figure 13:**
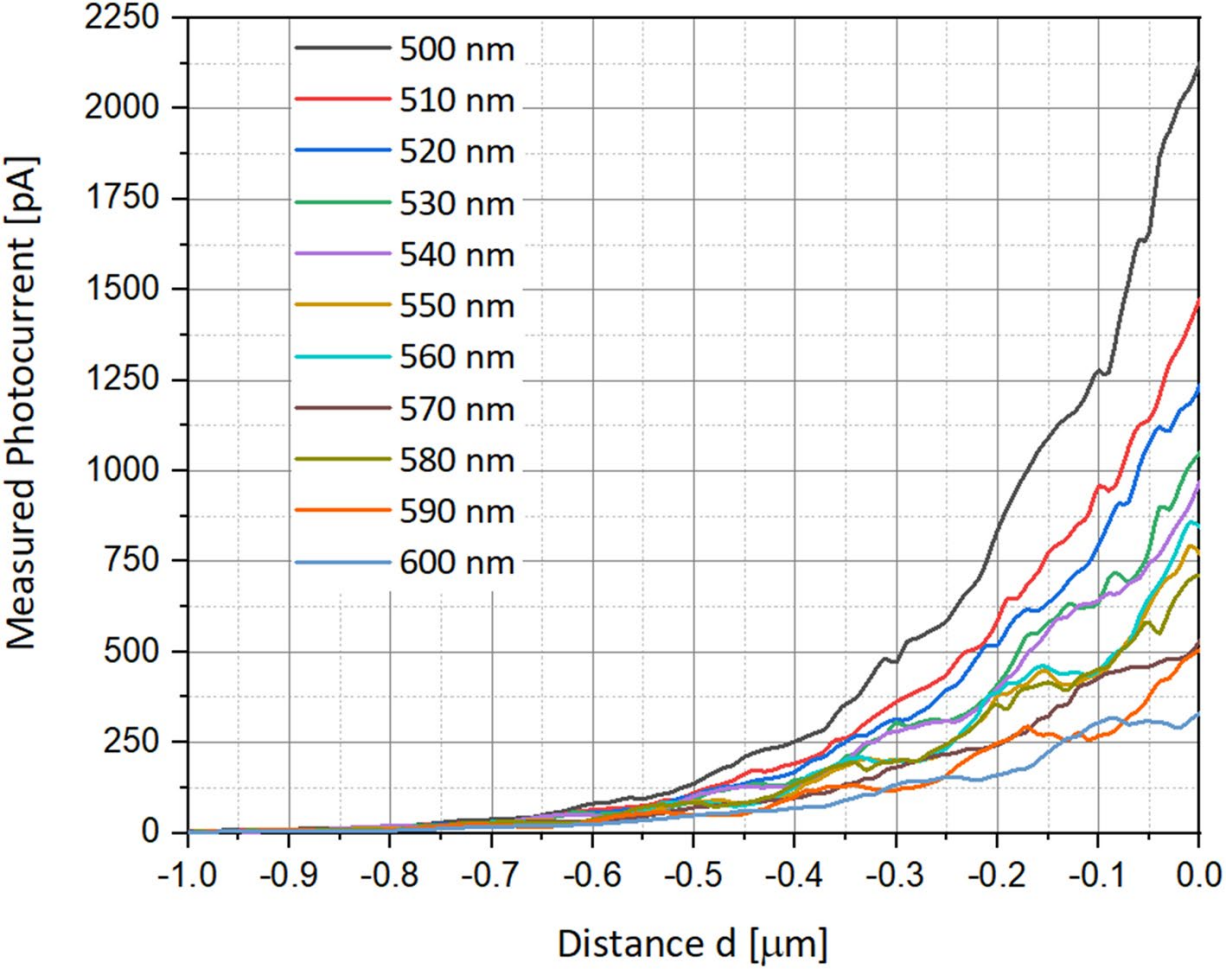
Measured photocurrent as function of the distance *d*, presented for several wavelengths between 500 and 600 nm. The distance $a$ is 5 nm, and the angle $\alpha$ is 45°. Chosen representative radius of the drilled aperture is 35 nm. All other parameters are presented in Table 1.

In order to better identify the optimal aperture diameter for such a scanning photodetector, series of simulations have been performed when varying the aperture diameter from 0 nm (e.g. filled device) to 150 nm (wavelength limit), by steps of 30 nm, as shown in Fig. 11. The simulation of the measured photocurrent as a function of the scanning distance *d*, and of the aperture radius *R*_*cylinder*_ is presented in Fig. 12. Based on different apertures (Fig. 11), the measured photocurrent curves present clear steps (Fig. 12) as a function of the radius. Except one curve, representing a photodetector sharing a radius of 0 nm (e.g. filled Schottky diode), all the other curves present the step-function phenomena. This can be explained by some diffraction and perhaps interference phenomena of the wave functions entering an aperture smaller than the wavelength. In Fig. 12, one can assume that the photocurrent values decrease with the increase of the aperture radii, since there is less silicon for light absorption, and since destructive interferences increase with the drilled aperture. In Fig. 13, one can assume that the photocurrent values decrease with the increasing wavelength, since we are working with sub-wavelength apertures. The higher is the wavelength, the smaller remains the aperture.

### Measured photocurrent as a function of the incident wavelength

Another necessary study was to check the influence of the incident wavelength on the measured photocurrent. For this purpose, simulations ran series of wavelengths, in a range of 500–600 nm (Fig. 13). The lower is the wavelength the higher is the measured photocurrent. In this experiment, the distance $\text{a}$ from the surface is varying from 5 to 50 nm, and the angle $\mathrm{\alpha }$ of the beam is 45°. The surface scanning is performed on a distance *d* of 1 µm.

### Stokes criteria in polarimeter tip

#### Multiple-apertures tip rationale

Moving forward with the drilled tip idea, an interesting and novel application to study would be to simultaneously generate four different holes, each sharing a different shape: one oval in X (*s* state polarizing), one in Y (*p* state polarizing) etc. such that the four photonic readouts forming the tip will be the four Stokes coefficients, in order to place the polarization state in the Poincare sphere. In such a way we can realize a Polarimeter on the tip! Since there are NSOM tips which are sensitive to polarization, but they do not perform any polarimetry measurement, we can do it with four different types of holes.

#### Stokes coefficients

The Stokes parameters are a set of values describing the polarization state of an electromagnetic radiation. Defined a long time ago by George Gabriel Stokes^20,21^, they represent the mathematically convenient alternative to the more common description of incoherent or partially polarized radiation in terms of its total intensity (I), (fractional) degree of polarization (p), and the shape parameters of the polarization ellipse. The effect of an optical system on the polarization of light can be determined by constructing the Stokes vector for the input light and applying Mueller calculus, to obtain the Stokes vector of the light leaving the system. The original Stokes paper was discovered independently by Perrin^22^ and by Chandrasekhar^23^, who named it as the Stokes parameters. The relationship of these Stokes parameters *S*_*0*_, *S*_*1*_,* S*_*2*_,* S*_*3*_ to the intensity and the polarization ellipse parameters is presented in below equations:

1 $${S}_{0}=I$$

2 $${S}_{1}=Ipcos2\psi cos2\chi $$

3 $${S}_{2}=Ipsin2\psi cos2\chi $$

4 $${S}_{3}=Ipsin2\chi $$

where $Ip$, $2\psi$ and $2\chi$ are the spherical coordinates of the three-dimensional vector of cartesian coordinates (${S}_{1}, {S}_{2}, {S}_{3}$). $I$ is the total intensity of the beam, and $p$ is the degree of polarization, constrained by $0\le p\le 1$. The factor of two before $\psi$ represents the fact that any polarization ellipse is indistinguishable from one rotated by 180°, while the factor of two before $\chi$ indicates that an ellipse is indistinguishable from one with the semi-axis lengths swapped accompanied by a 90° rotation. The phase information of the polarized light is not recorded in the Stokes parameters. The four Stokes parameters are sometimes denoted *I*, *Q*, *U* and *V*, respectively.

#### Scanning tip for nano-polarimetry

In order to measure the exact position of a polarization state on the Poincare sphere, one needs estimate the four Stokes parameters. Those parameters are defined as follows:

5 $$ S_{0} = P_{{0^{ \circ } }} + P_{{90^{ \circ } }} $$

6 $$ S_{1} = P_{{0^{ \circ } }} - P_{{90^{ \circ } }} $$

7 $$ S_{2} = P_{{ + 45^{ \circ } }} + P_{{ - 45^{ \circ } }} $$

8 $$ S_{3} = P_{{ + 45^{ \circ } ;\lambda /4}} - P_{{ - 45^{ \circ } ;\lambda /4}} $$

While the degree of polarization (DOP) can be computed out of the 4 Stokes parameters as:

9 $$DOP=\frac{\sqrt{{{S}_{1}}^{2}+{{S}_{2}}^{2}+{{S}_{3}}^{2}}}{{S}_{0}}$$

where $P_{{0^{ \circ } }}$ and $P_{{90^{ \circ } }}$ are the powers measured after linear horizontal and vertical polarizers respectively, $P_{{ + 45^{ \circ } }}$ and $P_{{ - 45^{ \circ } }}$ are the powers measured after linear $+ 45^{ \circ }$ and $- 45^{ \circ }$ polarizers respectively and $P_{{ + 45^{ \circ } ;\lambda /4}}$ and $P_{{ - 45^{ \circ } ;\lambda /4}}$ are the powers measured after $\lambda /4$ retarder and linear polarizers at $+ 45^{ \circ }$ and $- 45^{ \circ }$, respectively. $\lambda$ is the optical wavelength. The seven measurements are related to each other and actually it is enough to measure only four measurements: $P_{{0^{ \circ } }}$, $P_{{90^{ \circ } }}$, $P_{{ + 45^{ \circ } }}$ and $P_{{ + 45^{ \circ } ;\lambda /4}}$ from which the four Stokes parameters can extracted as:

10 $$ S_{0} = P_{{0^{ \circ } }} + P_{{90^{ \circ } }} $$

11 $$ S_{1} = P_{{0^{ \circ } }} - P_{{90^{ \circ } }} $$

12 $$ S_{2} = P_{{ + 45^{ \circ } }} + P_{{ - 45^{ \circ } }} = {2}P_{{ + 45^{ \circ } }} - S_{0} $$

13 $$ S_{3} = P_{{ + 45^{ \circ } ;\lambda /4}} - P_{{ - 45^{ \circ } ;\lambda /4}} = 2P_{{ + 45^{ \circ } ;\lambda /4}} - S_{0} $$

## Experimental realizations

**Figure 14:**
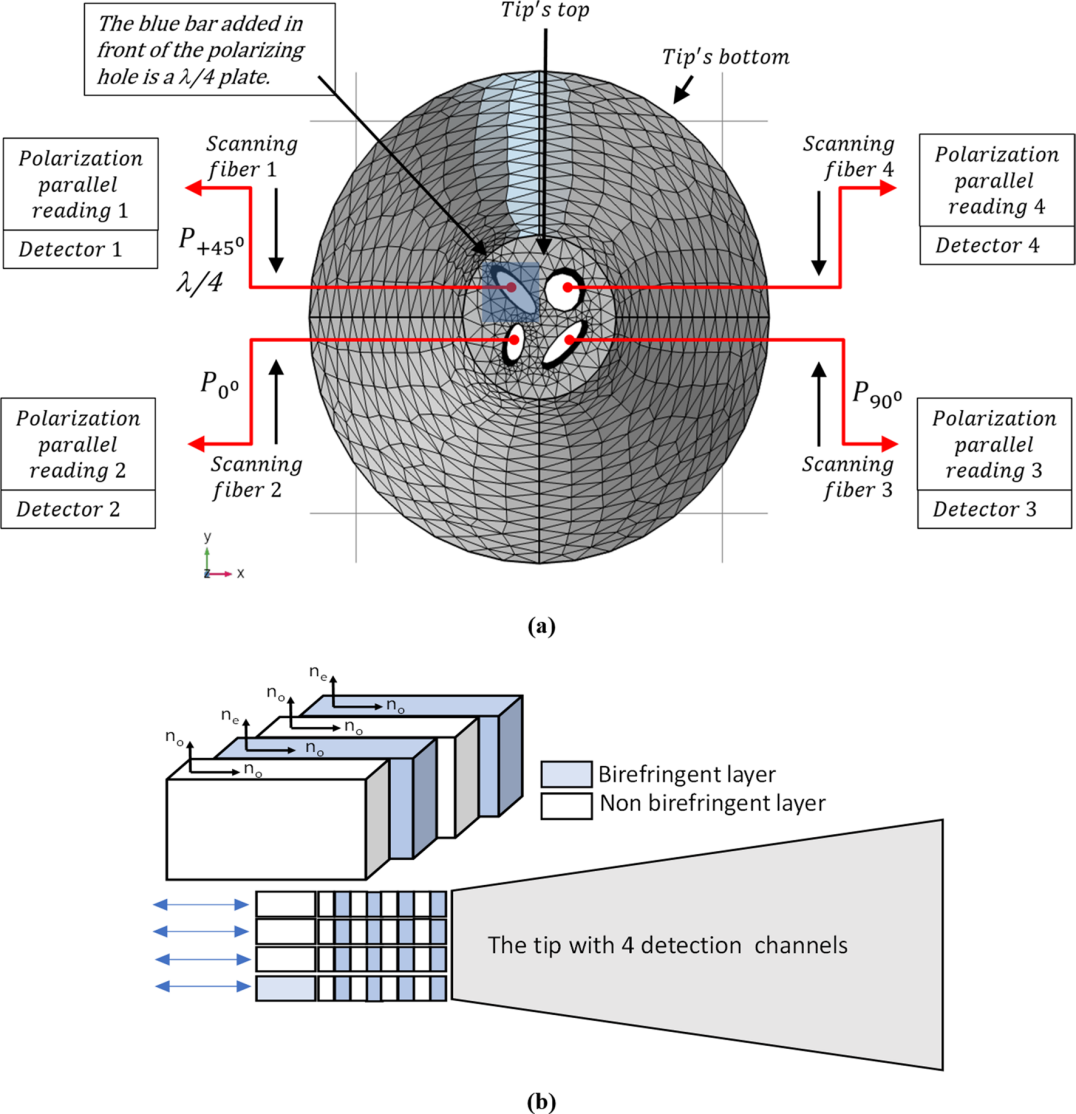
Experimental configurations. (**a**) First configuration of the four Stokes parameters measurement. (**b**) Second configuration based upon coating of periodic layers of birefringent material.

The four measurements expressed above, and which are required in order to extract the polarization state and to allocate the position of the polarization state on the Poincare sphere, can be measured in two possible configurations. One main configuration involves measuring the four parameters with the scanning nano tip while integrating into it the required components as depicted in Fig. 14a:

- $P_{{0^{ \circ } }}$, $P_{{90^{ \circ } }}$ and $P_{{ + 45^{ \circ } }}$ are realized by generating oval slits at the end of the tip. The tip is coated with metallic coating and by generating three different oval slits at $0^{ \circ }$, $90^{ \circ }$ and $+ 45^{ \circ }$ the three polarizers are achieved. The oval slits have one narrow dimension which is about $\lambda /10$ and one long dimension which is above one $\lambda$.
- $P_{{ + 45^{ \circ } ;\lambda /4}}$ can be realized by putting Yttrium Orthovanadate YVO4 (birefringent material) on the tip while the material is having sufficient width to realize $\lambda /4$ retarder (given its birefringence the required width will be about 2.5 $\lambda$). Later on, the signal being collected from the fiber is passed through a polarized at $+ 45^{ \circ }$.

All the four parameters are realized by properly fabricating the scanning tip and properly analyzing the collected intensities at the four detectors positioned on the other side of the scanning fiber.

Another proposed configuration for realizing the measurement includes coating of periodic layers of birefringent material on the edge of the scanning tip. Such coating could realize polarizer as well as $\lambda /4$ waveplate. The proposed configuration is seen in Fig. 14b. The coating could be obtained by putting several layers of YVO4 coated in the right crystal axes in respect to the axes of the incident light. One possible way of computing the structures of such layers could be by using Rouard’s method^24^.

The method is based on sectioning the grating into a set of thin layers, each treated as a layer with fixed index of refraction. Making use of Fresnel reflection and transmission coefficients enables the computation of a transfer matrix for each layer. For example, for the k’th layer, this matrix will have the form:

14 $$\left[\begin{array}{c}{R}_{k}\\ {S}_{k}\end{array}\right]=\frac{1}{{t}_{k}}\left[\begin{array}{cc}\mathrm{exp}(i{\varphi }_{k})& {r}_{k}\mathrm{exp}(i{\varphi }_{k})\\ {r}_{k}\mathrm{exp}(-i{\varphi }_{k})& \mathrm{exp}(-i{\varphi }_{k})\end{array}\right]\left[\begin{array}{c}{R}_{k+1}\\ {S}_{k+1}\end{array}\right]$$

with R_k_ and S_k_ being the forward and backward traveling fields respectively. r_k_ and t_k_ are the Fresnel reflection and transmission coefficients which satisfy:

15 $${r}_{k}=\frac{{n}_{k}-{n}_{k+1}}{{n}_{k}+{n}_{k+1}}$$

16 $${t}_{k}=\frac{2{n}_{k}}{{n}_{k}+{n}_{k+1}}$$

n_k_ and n_k+1_ are the refraction indexes in two sequential layers of the Bragg structure. As shown in Fig. 14b the Bragg structure has axial periodicity of two materials with different refraction indexes. Those two refraction indexes are denoted as n_k_ and n_k+1_. The phase φ_k_ represents the phase shift in the k’th section:

17 $${\varphi }_{k}=\frac{2{\pi n}_{k}}{\lambda }\delta {z}_{k}$$

where δz_k_ is the width of the section:

18 $$\delta {z}_{k}=\frac{\lambda }{2\left({n}_{k}+{n}_{k+1}\right)}$$

Multiplying all matrices yields the transfer matrix of the whole structure.

**Figure 15:**
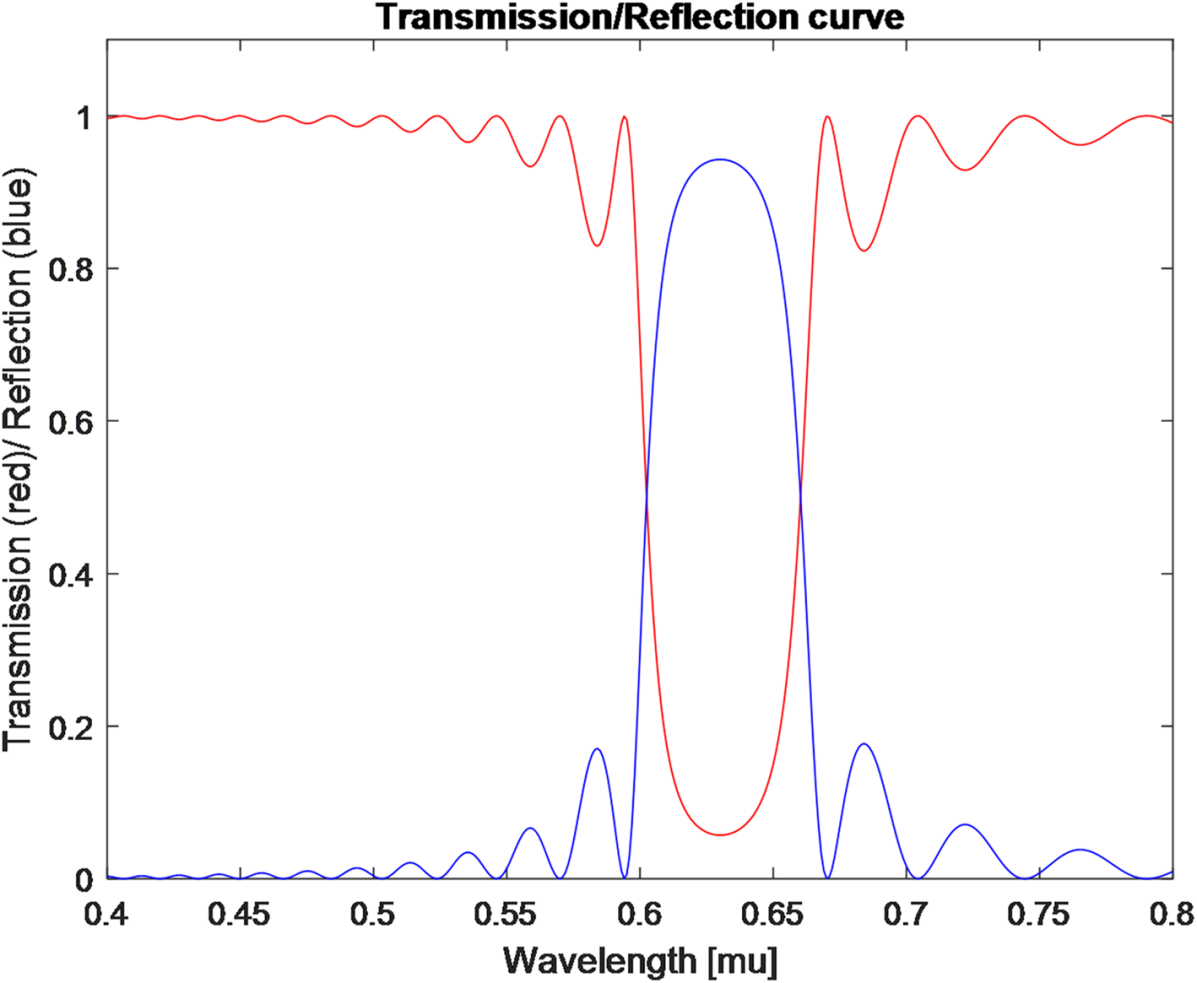
Simulation of a polarizer realized in multi payer Bragg structure deposited on the proposed tip.

For instance, in the simulation of Fig. 15 we used 20 layers of YVO4. At wavelength of 630 nm this material has refraction indexes of: n_o_ = 1.9929 and n_e_ = 2.2154. This means that the overall width of the proposed Bragg filter is:

19 $$  20 \times 2 \times \delta z_{k}  = 20 \times 2 \times {\lambda  \mathord{\left/ {\vphantom {\lambda  {2(n_{o}  + n_{e} )}}} \right. \kern-\nulldelimiterspace} {2(n_{o}  + n_{e} )}}\;3\;\mu m  $$

Thus, as seen in the figure, one polarization at wavelength of 630 nm is back reflected almost completely while the second will not see the periodic Bragg structure of the layers and will be transmitted through the generated structure. Thus, the simulation validates a realization of an optical polarizer that transmits only the horizontal polarization and back reflects the vertical polarization. The proposed configuration is seen in Fig. 14b. The bright blue is a birefringent material and the white blocks are non-birefringent material. Thus, as in the case of Fig. 14a the realization includes four channels with four detectors positioned as part of the proposed tip. However, in contrast to Fig. 14a no oval sub wavelength holes are needed. The three upper channels are the three polarizers of at 0°, 90° and + 45° while they are built as Bragg gratings while the vertical axis in which the Bragg is realized and where the polarizing effect occurs (the vertical axes is back reflected) is rotated at 0°, 90° and + 45° in respect to the vertical axis. The 4th channel has the Bragg based polarizer (the vertical axis of the schematic sketch is rotated at + 45°) while before that a birefringent material realizing $\lambda /4$ waveplate is deposited on the edge of the tip. This $\lambda /4$ wave plate is realized by deposing a birefringent material with width of:

20 $$\delta z=\frac{\lambda }{4\left({n}_{o}-{n}_{e}\right)}$$

which is approximately 0.708 μm for wavelength of 630 nm.

### COMSOL architecture and design

**Figure 16:**
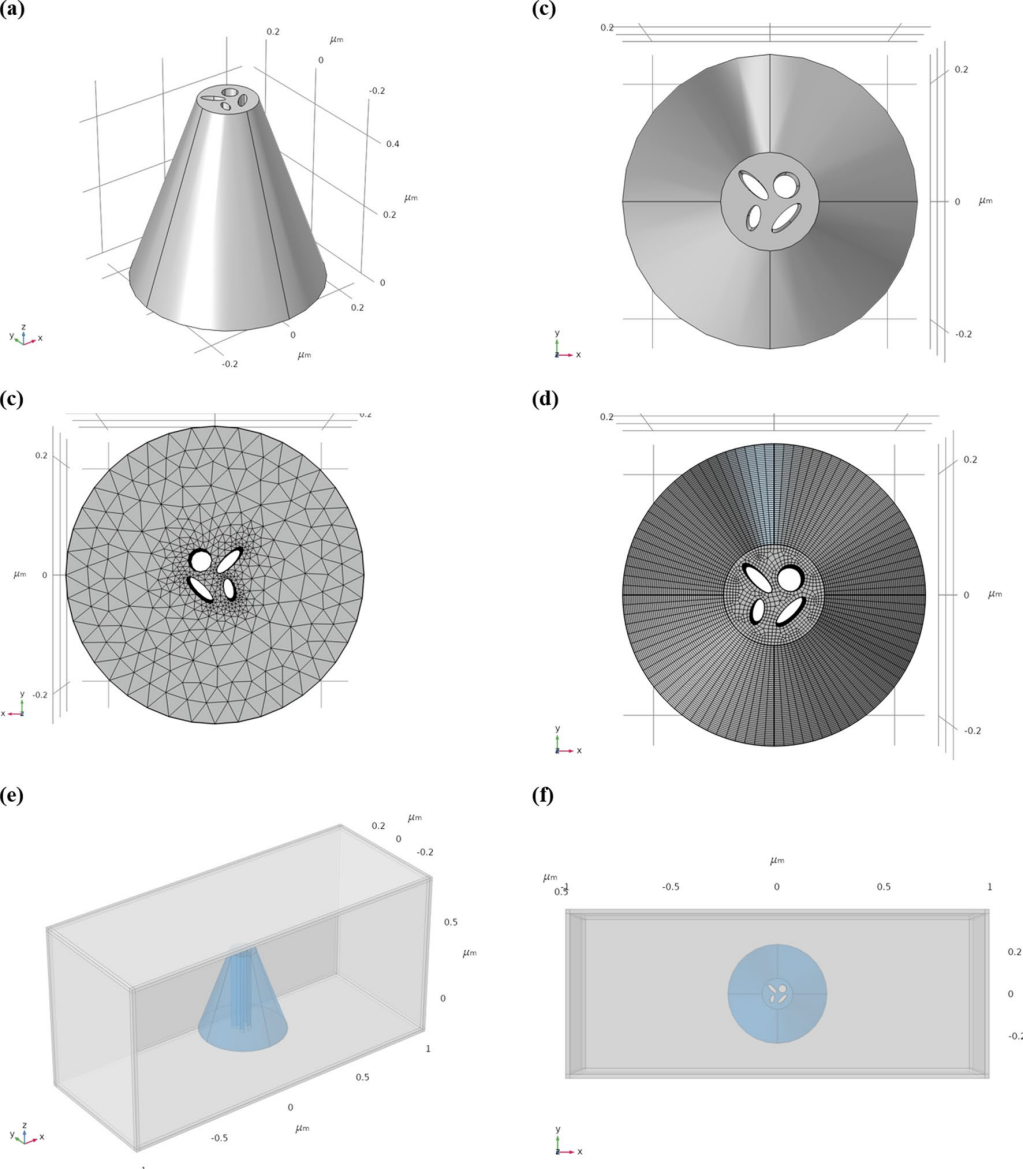
Schematic representations of the Polarimeter multiple-apertures proposed system. (**a**) Side view of the structure with the four apertures. (**b**) Top view of the structure with the four apertures. (**c**) Top view of the mesh structure with the four apertures using low density of finite elements. (**d**) Top view of the mesh structure with the four apertures using high density of finite elements. (**e**) Side view of the Polarimeter structure with the four apertures inside a simulation box. (**f**) Top view of the Polarimeter structure with the four apertures inside a simulation box.

In order to implement the four holes on the top of the tip, it was first necessary to design the structure of the nanoscale polarimeter, as well as the mesh of its different parts. Using again the Finite Elements Method (FEM), 2D and 3D silicon truncated-shaped conical photodetector has been designed, sharing subwavelength pin hole apertures. Then, at the top of the cone, four different shapes, round and ovals, have been selected to serve as the four photonic readouts, as presented in Fig. 16.

The necessity for an accurate mesh of finite elements is presented, when the density of the finite elements is varying from standard parts (Fig. 16c) to extra-fine parts (Fig. 16d). Since there is a need for accurate simulations, special boxes have been created (Fig. 16e,f).

### Preliminary experimental validation

#### Fabrication of tip-photodetectors

**Figure 17:**
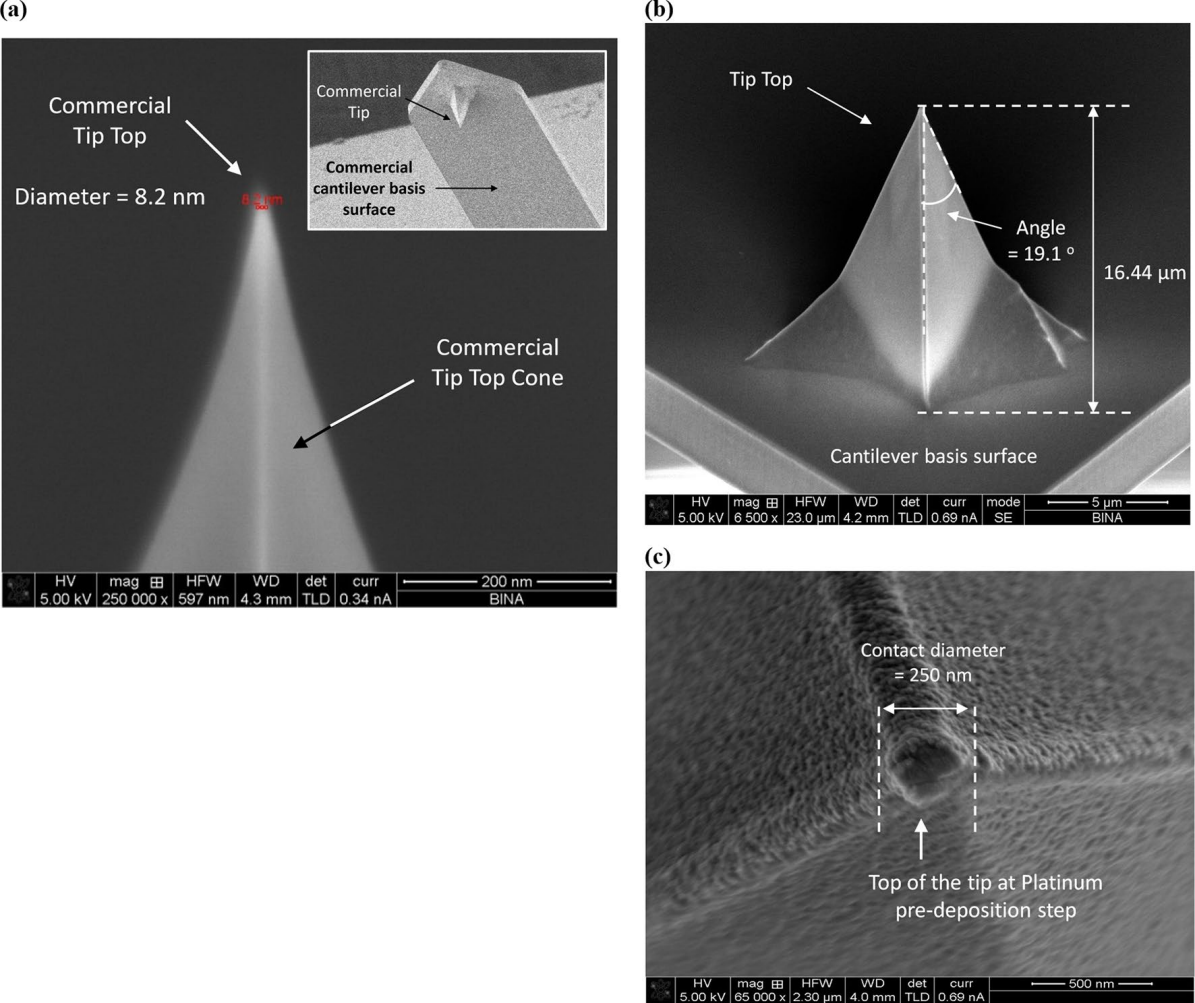
Presentation of selected SEM pictures of the AFM cantilever commercial starting material and of some of the fabrication steps towards its transformation to an active photodetector. (**a**) Upside view of the tip shape with an insert of the down side view of the cantilever and of the tip. (**b**) Tip height and basis. (**c**) Tip’s Top view at Platinum pre-deposition step.

In order to show preliminary experimental validation, we have fabricated an AFM-NSOM combined tip. We took a passive commercial silicon AFM tip which we transformed into an active photodetector at its top, such that the photonic readout could be provided directly. One of the main advantages of this AFM-NSOM dual-mode photodetector concept, is the fact that the fabrication process is quite simple, short (few hours overall), and starts from a commercial tip. The six-step rapid process has been largely presented in the past^15^, when starting from standard commercial (FESP-V2 model from Bruker)^25^ AFM silicon-based tips. Moreover, additional studies of such tip-photodetector have been presented as well^26^. The profile and the dimensions of the commercial cantilever and of the commercial tip are respectively presented in Fig. 17a–c.

**Figure 18:**
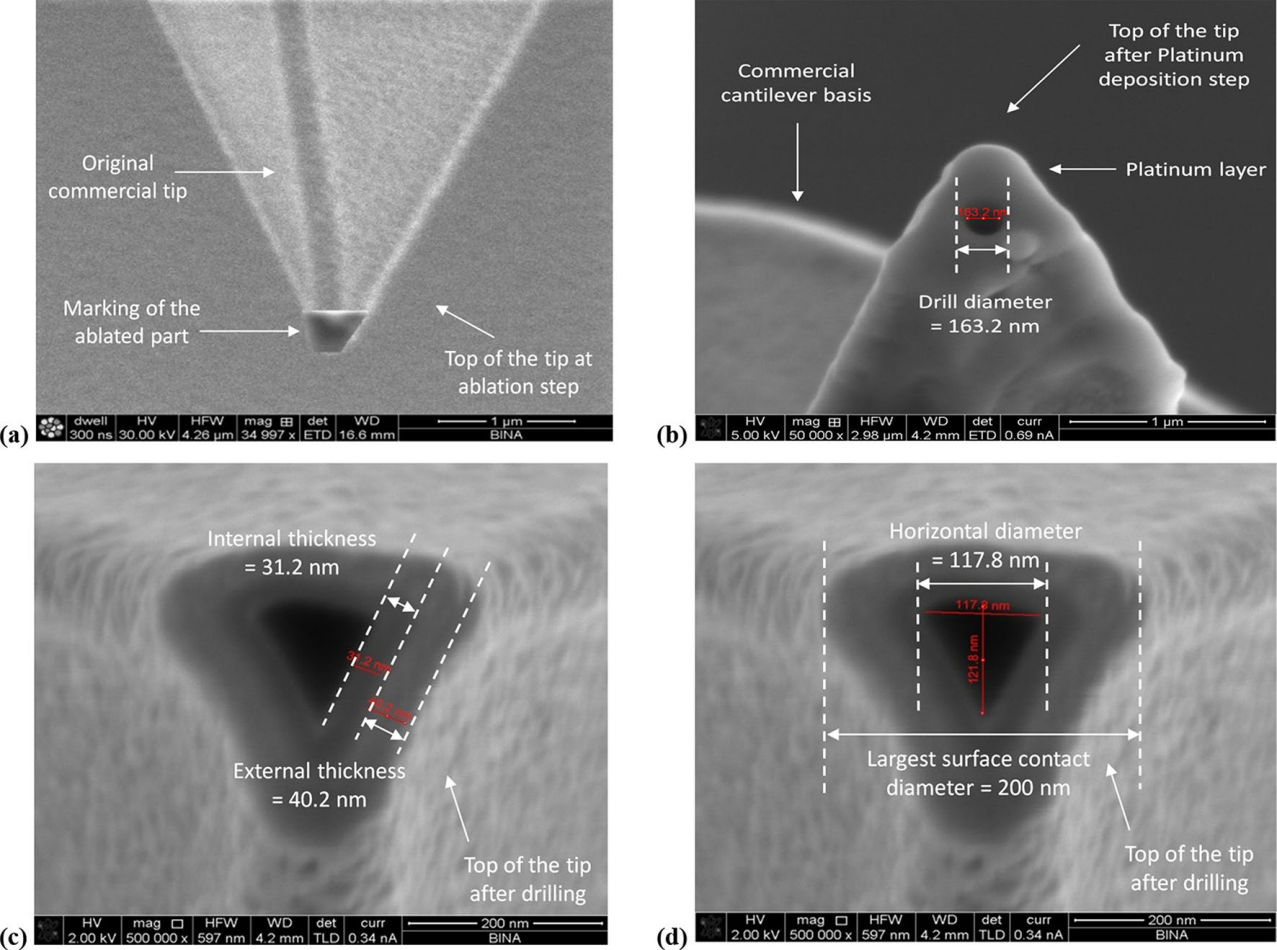
SEM pictures of the tip after drilling and platinum deposition needed to obtain conductance of the fabricated photo detector. (**a**) Tip after FIB ablation. The tip was cut with a Ga+ ion beam current of 2.7 nA, accelerated by a voltage of 2 kV fixed on the FIB device. (**b**) Tip after platinum deposition. The platinum, coming from the vapor precursor, was deposited by the Ga+ ion beam on the top of the probe. (**c**) Upside view of a truncated tip with the layers’ thicknesses. The depth of the drilling was 400 nm through the platinum layer, and a conical hole was obtained. (**d**) Upside view of a truncated tip with the internal diameters of 117 nm and 121 nm, which approximately correspond to radii of 60 nm, as presented in Fig. 12.

In Fig. 18 we show now the next three fabrication steps, after processing them with a Focused Ion Beam (FIB) system: the ablation of the tip top (Fig. 18a), the Platinum deposition needed to generate the conductance (Fig. 18b), and the drilling of the cone (Fig. 18c,d). These three steps were all processed using the Field Electron and Ion company (FEI) Helios 600 FIB system. Several important considerations came up in the choice of this specific equipment: First, its main advantage remains its dual-beam component, enabling combined Scanning Electron Microscopy (SEM) and FIB technologies, in addition to large diversity in gas chemistries, detectors, and manipulators. The second reason is more related to ions’ type used in the process. In fact, there are two main types of ions used in such a FIB: Ga+ ions and He ions. For this specific drilling process, it appears that the He ions beam is not strong enough to enable a well-done truncated tip, and the Ga+ ions become necessary. On the other hand, there is a concern when using Ga+ ions, since they are capable to cause a significant degradation of the initial silicon crystalline structure, and as a consequence, to affect the electro-optic measurements. Since the Ga+ ions are implanted into the Silicon structure during the FIB processing part, and since they theoretically can be be located in the critical area where there is a need to detect a photo current, they may influence the Signal-to-Noise Ratio (SNR). This concern is reinforced when compared to the implantation process for regular ions, there is no annealing step or any thermal post-recovery process after the drilling stage. In order to assure the quality of the electrode and of the Schottky contact, several checks were performed, and the parts were found functional.

#### Non-altered AFM spatial high resolution

It is well known that the high spatial resolution of the atomic force microscopy depends on the probe size, i.e. the contact surface. Generally, standard AFM probes share a tip radius of less than 10 nm. A priori, when a tip is blunt, it becomes difficultly possible to measure the details on the topography of a substrate in picometer or nanometer ranges. A priori, one could argue that our AFM-NSOM probe is blunt, when the contact surface is ten times larger than a commercial tip’s contact diameter. In order to assure that the AFM measurement technique is still relevant, we already presented preliminary experimental results^15^ for surface topography of materials scanned with these blunt AFM-NSOM probes, in order to show how the AFM imaging capability is not altered.

#### Multi-truncated tips

Looking forward, the next step, which could not be performed yet due to the undefined COVID-19 confinement period, would be to perform the ablation of the tip at different heights, this in order to enable larger contact surfaces in which the FIB will drill this time several apertures on the same plane, as presented above in Figs. 16 and 18. This part will consist in progressive work, with series of samples, sharing different numbers of apertures (from two to four), and different diameters of apertures. Such proposed tip, combining high spatial resolution together with polarimetric capability, is very novel, and has better been demonstrated before. Moreover, it has high applicability in scanning microscopy to be used for improved mapping of functionality of nano photonics devices. The novelty is related to the fact the proposed probe, when scanning nano photonic devices, could provide topography readout together with electro-magnetic intensity distribution, as in NSOM, but together with its polarization state in every sampled point. Such capability yields functionality significantly enhancing the characterization abilities of nano devices, and better understanding their operability. The added value of this specific paper is the capability of combining three modalities together, allowing not only to readout the intensity with nanometric resolution, but also to extract its polarization state.

## Conclusions

In this article, a new concept of improved scanning system was presented. The new system has been built on standard Atomic Force Microscope (AFM) cantilever, in order to serve as a triple-mode scanning system, sharing complementary scanning mechanical topography (AFM), optical data analysis (NSOM), and polarization states (four multiple apertures as four Strokes criteria). This win–win combining probing system enabling light collection through a very sensitive silicon-based photo-detector, has been designed and simulated using Comsol Multi-Physics software package, and consists in a Platinum-Silicon drilled conical photodetector, sharing subwavelength apertures. It has been processed using advanced nanotechnology tools on a commercial silicon cantilever. After a comparison study of drilled versus filled tips advantages, and of several optics phenomena such as interferences, the article presented the added value of multiple-apertures scanning tip for nano-polarimetry. Such advanced system will enable crossing information of mechanical, optical and polarization results during the samples’ scanning process, and thorough and more accurate analysis of the observed results. The importance of using a drilled photodetector when compared to a filled one was discussed. Unfortunately, due to the COVID-19 unlimited unknown confinement period, the next steps will need to be part of an additional research and publication (Supplementary Information).

## Supplementary information

Supplementary Information.

## References

[1] Betzig E, Lewis A, Harootunian A, Isaacson M, Kratschmer E (1986). Near-field scanning optical microscopy (NSOM), development and biophysical applications. Biophys. J..

[2] Grabar KC, Brown KR, Keating CD, Stranick SJ, Tang SL, Natan MJ (1997). Nanoscale characterization of gold colloid monolayers: A comparison of four techniques. Anal. Chem..

[3] Lowman GM, Daoud N, Case RM, Carson PJ, Buratto SK (2001). Local energy transfer in self-assembled polyelectrolyte thin films probed by near-field optics. Nano Lett..

[4] Jiang R-H, Chen C, Lin D-Z, Chou H-C, Chu J-Y, Yen T-J (2018). Near-field plasmonic probe with super resolution and high throughput and signal-to-noise ratio. Nano Lett..

[5] Ozcan A, Cubukcu E, Bilenca A, Crozier KB, Bouma BE, Capasso F, Tearney GJ (2006). Differential near-field scanning optical microscopy. Nano Lett..

[6] Kerimo J, Adams DM, Barbara PF, Kaschak DM, Mallouk TE (1998). NSOM investigations of the spectroscopy and morphology of self-assembled multilayered thin films. J. Phys. Chem. B.

[7] Dutta AK, Vanoppen P, Jeuris K, Grim PCM, Pevenage D, Salesse C, De Schryver FC (1999). Spectroscopic, AFM, and NSOM studies of 3D crystallites in mixed Langmuir−Blodgett films of N,N′-Bis(2,6-dimethylphenyl)-3,4,9,10-perylenetetracarboxylic diimide and stearic acid. Langmuir.

[8] Atomic Force Microscopy Market 2018, Nanonics Ltd. Internal study.

[9] Harris, C. M. The Saga of AFM, a journey into a hot analytical market. *Anal. Chem*. 627–635 (2001). 10.1021/ac012631x 11721950

[10] Harris, C. M. Shedding light on NSOM. *Anal. Chem*. 223–228 (2003). 10.1021/ac0313264 12751532

[11] Arora S, Sumati A, George PJ (2012). Design of mems based microcantilever using comsol multiphysics. Int. J. Appl. Eng. Res..

[12] Lu F, Jin M, Belkin MA (2014). Tip-enhanced infrared nanospectroscopy via molecular expansion force detection. Nat. Photonics.

[13] Stern L, Desiatov B, Goykhman I, Lerman GM, Levy U (2011). Near field phase mapping exploiting intrinsic oscillations of aperture NSOM probe. Opt. Express.

[14] Zhao Y, An KH, Chen S, O’Connor B, Pipe KP, Shtein M (2007). Localized current injection and submicron organic light-emitting device on a pyramidal atomic force microscopy tip. Nano Lett..

[15] Karelits M, Lozitsky E, Chelly A, Zalevsky Z, Karsenty A (2019). Advanced surface probing using dual-mode NSOM-AFM silicon-based photo-sensor. Nanomaterials.

[16] Comsol Multi-Physics Software Package, https://www.comsol.com/.

[17] Karsenty A, Mandelbaum Y (2019). Computer algebra challenges in nanotechnology: Accurate modeling of nanoscale electro-optic devices using finite elements method. Math. Comput. Sci..

[18] Karsenty A. & Mandelbaum, Y. computer algebra in nanotechnology: modelling of nano electro-optic devices using finite element method (FEM). In *Proceeding of ACA 2017 23rd Conference on Applications of Computer Algebra, Session 6: Computer Algebra for Applied Physics*, 138, Jerusalem (July 17–21, 2017).

[19] Karelits M, Mandelbaum Y, Chelly A, Karsenty A (2017). Electro-optical study of nanoscale Al–Si truncated conical photodetector with subwavelength aperture. J. Nanophotonics.

[20] Stokes GG (1852). On the composition and resolution of streams of polarized light from different sources. Trans. Cambridge Philos. Society.

[21] Chandrasekhar S (1960). Radiative Transfer.

[22] Perrin F (1942). Polarization of light scattered by isotropic opalescent media. J. Chem. Phys..

[23] Chandrasekhar S (1947). The transfer of radiation in stellar atmospheres. Bull. Am. Math. Society.

[24] Weller-Brophy LA, Hall DG (1985). Analysis of waveguide gratings: Application of Rouard’s method. J. Opt. Society Am. A (JOSA A).

[25] Bruker AFM Probes, https://www.brukerafmprobes.com/p-3897-fesp-v2.aspx.

[26] Karelits M, Mandelbaum Y, Zalevsky Z, Karsenty A (2019). Time-spectral based polarization-encoding for spatial-temporal super-resolved NSOM readout. Nat. Sci. Rep..

