# Supplementary figures and images for: Nano polarimetry: enhanced AFM-NSOM triple-mode polarimeter tip
Source: Sci Rep. 2020 Oct 1;10:16201. doi: 10.1038/s41598-020-72483-9 (PMC7529902; doi:10.1038/s41598-020-72483-9)

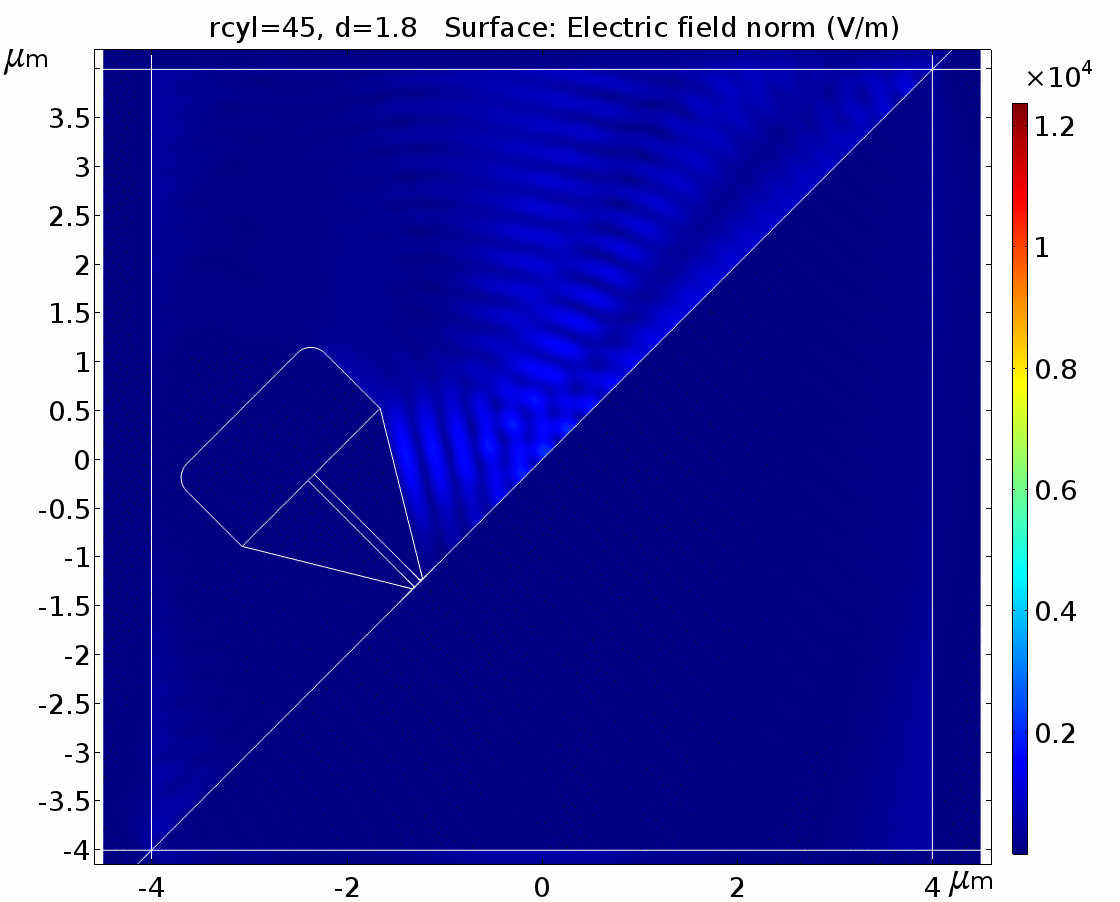

Supplement: Supplementary file 1 — Supplementary Information. [file 41598_2020_72483_MOESM1_ESM.gif]
